# Supplementary material for: Southern Ethiopian skilled birth attendant variations and maternal mortality: A multilevel study of a population-based cross-sectional household survey
Source: PLOS Glob Public Health. 2023 Dec 27;3(12):e0002466. doi: 10.1371/journal.pgph.0002466 (PMC10752526; doi:10.1371/journal.pgph.0002466)
Supplement: Attachment — Submitted filename: Response to Reviewers.docx [file pgph.0002466.s003.docx]

**Response to Reviewers**

**PGPH-D-23-01804**

**Southern Ethiopian skilled birth attendant variations and maternal mortality: a multilevel study of a population-based cross-sectional household survey**

**PLOS Global Public Health**

**Point by point response to reviewers’ and editor comments**

The authors would like to thank the reviewers and editor for their time, suggestions and valuable comments. We have carefully addressed all the comments and suggestions. The corresponding changes made in the revised manuscript are summarized in our response below.

**Response to Reviewer #1 comment**

**Comment 1.1**

The objectives of the study are many and important but need to present more clearly.

**Response:** We would like to thank the reviewer for pointing out this issue. We revised the objectives to make them clearly understood. (Lines 97-101).

**Comment 1.2**

The methodology comprising the study population, the sampling design, data analysis, and ethical consideration are comprehensively presented in the manuscript.

**Response:** We would like to thank the reviewer for appreciation and for mentioning the strong points of methodology section.

**Comment 1.3**

The section in the methods that I think the authors need to address is the sample size presented. In the abstract, 3191 women were said to have been interviewed but in the sample determination, figures such as 768 and 8880 are also talked about. There appears to be contradiction here and so, please, reconcile and be consistent.

**Response:** We appreciate the reviewer mentioning this issue. This study is part of a larger study aiming at measuring maternal mortality in Sidama National Regional State, southern Ethiopia. The study population for the current study is women who had given birth in the past two years preceding the survey, which is a subset of the study population of the larger study. We calculated the minimum sample needed for the current study, which is 768. By adding 10% of non-response the minimum sample needed for this study would be 845 women. The 8880 are the households needed for the larger study. In these 8880 households, we found 3191 women who had given birth in the past two years, and we included them all in our study. We modified the sentence in abstract as follow “A total of 3191 women who had given birth in the past two years and resided in 8880 households sampled for the associated maternal mortality household survey were interviewed” (Lines 34-36). We also modified the sentence under the subheading of sample size determination (Line 221).

**Comment 1.4**

In the introduction, the authors seem to be reporting results of their study in lines 84-91. Please, check and reserve your findings to the results section.

**Response:** We would like to express our thanks to the reviewer for mentioning this important point. As it has been said, it seems reporting results in the introduction section. This is part of a larger study that assessed maternal mortality in the region using two different methods. Therefore, the results reported here are from these papers, which have already been published. In the result and discussion sections, we described our examination of the association of the use of skilled birth attendant services with maternal mortality using maternal mortality study findings, which used a five-year recall of birth and pregnancy outcomes.

**Comment 1.5**

The policy recommendations appear to sound like authorizing or commanding instead of advocacy. Please, check.

**Response:** Thanks for mentioning this. Yes, it’s true that the recommendations seem commanding rather than advocating for the concerned bodies. We modified the wording of the sentence (lines 471-484) and (lines 486-491).

**Response to additional comments in the PDF document**

**Comment**: Means of travel in abstract section

**Response:** edited (line 38)

**Comment**: Add exclusion or inclusion criteria in method section

**Response:** We added two sentences describing exclusion criteria (lines 135-137).

**Comment**: TBA?

**Response:** Both trained and untrained TBAs are considered unskilled and hence we expanded the description (lines 149-150)

**Comment**: sample size

**Response:** described above. We also described (edited) in the abstract section

**Comment**: Source of maternal mortality ratio (MMR) data which is mentioned in result section

**Response:** The MMR mentioned in the result section was obtained from the larger maternal mortality study, of which the current study is part. We used this data to examine the association between the use of skilled birth attendant services and maternal mortality. Under the subheading of data analysis, we mentioned the source of data for MMR and cited the reference (Line 248: Ref [11]).

**Comment**: Recommendations seems commanding rather than advocating

**Response:** described above. We edited the wording of the sentences under the subheadings of policy implications and conclusions

**Response to Reviewer #2 comment**

**Comment 2.1: Abstract:**

The abstract is well written. It summarizes the important aspects of the study well. However, to comply with the journal guidelines, subheadings may be eliminated.

**Response:** We would like to thank the reviewer for the appreciation and suggestion to remove the subheadings from the abstract. Hence, we deleted the subheadings accordingly (see lines 20, 25, 33, and 45) and submitted the revised abstract.

**Comment 2.2: Introduction**

The introduction gives a good preamble to the research topic. It sets a good background for the reader to understand this research topic. However, it would be nice to describe the setting of Sidama national region including the physical, social, economic structures as this helps the reader to understand the research context better.

**Response:** We appreciate the reviewer mentioning an important point. We elaborated the description of the study area by adding information regarding population, main source of income, administrative structures, and health facilities existing in the region (lines 104–119).

**Comment 2.3: Methods**

The methods are described sufficiently for the research to be reproduced.

**Response:** We would like to thank the reviewer for appreciation and for mentioning the strong points of methods section.

**Comment 2.4: Results**

The results are well presented. The effect of larger sampling units on the results was accounted for, which is good. However, the number of women interviewed is a lot higher than the proposed sample size, is there an explanation for this?

**Response:** Thanks for mentioning this point. As we mentioned earlier, this study is part of a larger study in the Sidama region aimed at measuring maternal mortality. The study population of this study was a subset of the population of the larger study. Initially, we calculated the minimum sample size needed for this study. When we did the actual data collection, we included all women who had given birth in the past two years prior to the study and who resided in the 8880 households sampled for the larger study.

**Comment 2.5: Results**

The data in support of these has not been provided yet and therefore, the results cannot be proven.

**Response:** Thanks for mentioning this. We have already deposited our data to data repository and we will share the link to the public data repository upon notification of formal acceptance of the manuscript.
